# Genetic variation of *pfhrp2* in *Plasmodium falciparum* isolates from Yemen and the performance of HRP2-based malaria rapid diagnostic test

**DOI:** 10.1186/s13071-015-1008-x

**Published:** 2015-07-22

**Authors:** Wahib M. Atroosh, Hesham M. Al-Mekhlafi, Adel Al-Jasari, Hany Sady, Ahmed K. Al-Delaimy, Nabil A. Nasr, Salwa Dawaki, Awatif M. Abdulsalam, Init Ithoi, Yee Ling Lau, Mun Yik Fong, Johari Surin

**Affiliations:** Department of Parasitology, Faculty of Medicine, University of Malaya, 50603 Kuala Lumpur, Malaysia; Department of Microbiology and Parasitology, Faculty of Medicine and Health Sciences University of Aden, Aden, Yemen; Azal National Research Centre, Azal University for Human Development, 447 Sana’a, Yemen; Department of Parasitology, Faculty of Medicine and Health Sciences, Sana’a University, 1247 Sana’a, Yemen; National Malaria Control programme, Ministry of Public Health and Population, Sana’a, Yemen

**Keywords:** Malaria, *Plasmodium falciparum*, Rapid diagnostic test, *Plasmodium falciparum* histidine-rich protein 2, Yemen

## Abstract

**Background:**

The genetic variation in the *Plasmodium falciparum* histidine-rich protein 2 (*pfhrp2*) gene that may compromise the use of *pfhrp2-*based rapid diagnostic tests (RDTs) for the diagnosis of malaria was assessed in *P. falciparum* isolates from Yemen.

**Methods:**

This study was conducted in Hodeidah and Al-Mahwit governorates, Yemen. A total of 622 individuals with fever were examined for malaria by *CareStart*™ malaria HRP2-RDT and Giemsa-stained thin and thick blood films. The *Pfhrp2* gene was amplified and sequenced from 180 isolates, and subjected to amino acid repeat types analysis.

**Results:**

A total of 188 (30.2 %) participants were found positive for *P. falciparum* by the RDT. Overall, 12 different amino acid repeat types were identified in Yemeni isolates. Six repeat types were detected in all the isolates (100 %) namely types 1, 2, 6, 7, 10 and 12 while types 9 and 11 were not detected in any of the isolates. Moreover, the sensitivity and specificity of the used PfHRP2-based RDTs were high (90.5 % and 96.1 %, respectively).

**Conclusion:**

The present study provides data on the genetic variation within the *pfhrp2* gene, and its potential impact on the PfHRP2-based RDTs commonly used in Yemen. *CareStart*™ Malaria HRP2-based RDT showed high sensitivity and specificity in endemic areas of Yemen.

**Electronic supplementary material:**

The online version of this article (doi:10.1186/s13071-015-1008-x) contains supplementary material, which is available to authorized users.

## Background

Malaria is still a major public health problem in Yemen, with almost 66 % of the population living in areas that suffer from stable malaria transmission [1]. *Plasmodium falciparum* is the predominant species and was responsible for almost 99 % of malaria cases in Yemen during 2012, a large number of which consisted of drug-resistant *P. falciparum* parasites [2, 3]. Among 17 countries with malaria-endemic areas in the Middle East and Eurasia region; Pakistan, Afghanistan and Yemen account for more than 99 % of the 56,000 regional deaths due to malaria [4].

The national malaria control programme in Yemen (NMCP) has achieved substantial success in controlling local cases of malaria, achieving a significant reduction in the number of malaria cases, dropping from 900,000 cases in the early 2000s to around 150,000 cases by 2013 [1]. However, Yemen is still classified among areas of high malaria transmission, making it the only country in the Arabian Peninsula and greater Middle Eastern region that is still plagued with malaria to the extent that residents still suffer from considerably high mortality and morbidity rates [2]. Imported malaria cases are still reported in neighbouring countries, threatening the malaria control and elimination programmes in the region. For instance, 2788 malaria cases were diagnosed in southern Saudi Arabia between 2011 and 2012, with about 97 % of the cases having been identified as originating outside the country, particularly from the Tehama region, a Yemen bordered area [5].

Early and accurate diagnosis of malaria, along with prompt treatment, are essential to reduce the burden of the disease worldwide. Rapid diagnostic tests (RDTs) have been widely used for the diagnosis of malaria and become an indispensable tool for malaria case-management, control and elimination worldwide, especially in rural endemic areas without laboratory access [6, 7]. Besides the affordability and shorter turnover time of RDT-based diagnosis, significant reductions in the over-prescription of antimalarials have been reported when RDTs are introduced in presumptive treatment settings, especially with the new policy of using the expensive artemisinin-combination therapy (ACT) as the first line treatment for uncomplicated falciparum malaria infection [8]. However, recent studies revealed that the sensitivity of RDTs could be compromised due to genetic polymorphism of the parasite PfHRP2 antigens, particularly with regards to certain amino acid repeat types, causing false-negative results when using the HRP2-based RDT to diagnose *P. falciparum* malaria [9–11].

In Yemen, PfHRP2-based RDTs have been implemented by the NMCP in 2009, and are being used exclusively for malaria active case detection (ACD) targeting only falciparum malaria infections [12]. However, data on the genetic variation of the *pfhrp2* are not available. Hence, the present study aims to investigate the genetic variations of the *pfhrp2* gene in malaria isolates from the Hodeidah and Al-Mahwit governorates, Yemen (areas with high malaria endemicity) and the possible impact of this variation on the efficacy of the currently used *pfhrp2*-based RDTs. The present study is the first to provide data on the genetic variation of PfHRP2 in Yemen, and therefore has the potential to impact on control strategies and efforts to eradicate malaria from Yemen and the Arabian Peninsula.

## Methods

### Study area

An active case detection survey targeting individuals with fever suspected of having a malaria infection was carried out in some malaria endemic districts of the Tehama region in both the Hodeidah and Al-Mahwit governorates, Yemen. The survey was carried out from March to May 2014, during the malaria transmission season. Districts with high malaria endemicity; namely AdDahi, Al-Marawiah, and Bajil from Hodeidah, and Khamis Bani Saad from Al-Mahwit, were selected based on the national malaria records of 2010–2013 provided by NMCP.

**Fig. 1:**
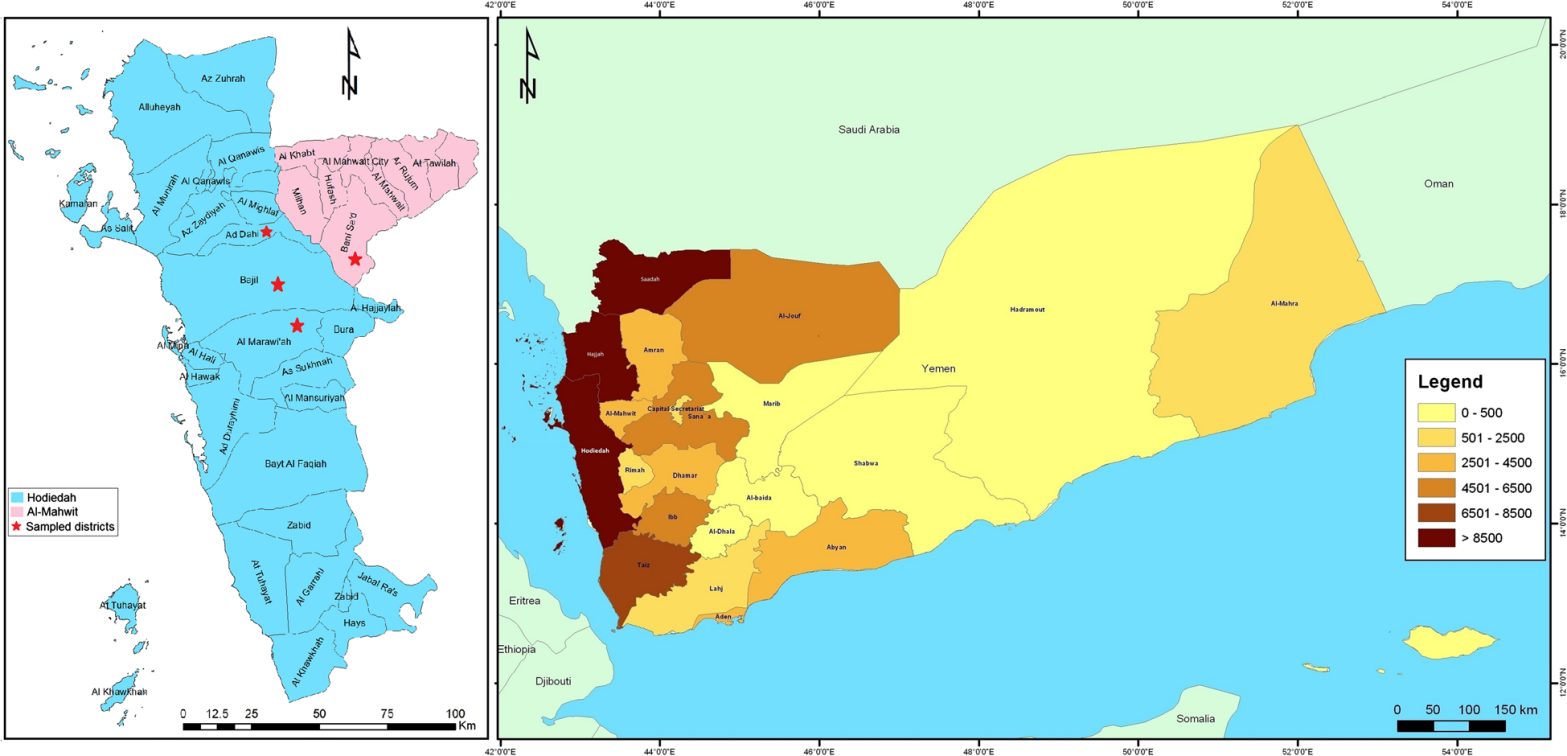
A geographic map showing study area (Hodeidah and Al-Mahwit governorates) and the distribution of malaria in Yemen according to incidence in 2012

The Hodeidah governorate (14.46° North, 43.15° East) is located in the Tehama region in the western part of Yemen, about 226 km from Sana’a, the capital of Yemen. It is a coastal area located along the Red Sea, covering a total area of 117,145 km^2^ with a total population of 2.16 million [13]. Al-Mahwit governorate (16.25° N, 44.717° E) is located between Hodeidah and Sana’a (about 111 km west of Sana’a), covering a total area of 2858 km^2^ with a total population of 597,000 people [13]. The climate of the selected districts is a combination of tropical monsoon with occasional rains in the summer and dry weather in winter, with a mean rainfall of 200 mm/year. The mean temperature is 37.5 °C in summer and 24 °C in winter, with humidity ranging between 70 and 90 %. Malaria is highly prevalent in Tehama region with high transmission peaking between January and March each year. Figure 1 shows the study area and the distribution of malaria burden in Yemen in 2012.

#### *Plasmodium falciparum* isolates

A total of 622 individuals with fever were recruited to this study and examined for malaria. Finger prick blood samples were collected from participants and tested using the RDT (*CareStart*™ Malaria HRP2, Cat no. G0141, Access Bio, Inc, USA), and for preparing both thick and thin blood films. Filter paper blood spots were also collected from each participant on 3MM Whatman® filter paper (Whatman International Ltd., Maidstone, England), and kept in clean, dry, well-sealed aluminum pouches with desiccated silicon bags for molecular analysis. Blood films were stained with 5 % of buffer-diluted Giemsa stain for 30 min and were examined microscopically for the presence of malaria parasites. All RDT tests were performed and interpreted by trained and skilled laboratory personnel from the NMCP following the manufacturer’s instructions. Briefly, one purple line in the control line position interpreted negative for falciparum malaria and two purple lines at the test line position along with the control line were to be defined as positive. In case the control line did not appear, the result was considered invalid and the test was repeated.

For positive slides, parasite species and stages were reported and parasitaemia (parasite density) was determined by counting only the asexual stages against 300 white blood cells (WBC) and then multiplied by 25; assuming the average of total WBC count of individuals equal to 7500 cells per μl of blood. The level of parasitaemia was graded as low (<1000 parasites/μl of blood), moderate (1000–9999 parasites/μl of blood) and severe (≥10,000 parasites/μl of blood). A double check for malaria microscopy was performed by two senior malaria microscopists; slides were examined twice and the average parasitemia per microlitre of blood was recorded for every microscopy positive slide. Genomic DNA was extracted from the filter paper blood spots and subjected to *pfhrp2* amplification using conventional single-run PCR.

The protocol used in this study was approved by the Ethics Committee of the University of Malaya Medical Centre, Malaysia (Ref. 974.19). The protocol was also approved by the Ministry of Health and Population, in conjunction with the National Malaria Control Programme in Yemen. Written and signed or thumb-printed informed consents were taken from adult participants and parents or guardians on behalf of their children before starting the sample collection; these procedures were also approved by the ethics committees. RDT-positive participants were treated with artemisinin combination therapy (artemisinin + sulfadoxine/pyrimethamine) according to the national malaria treatment policy, Ministry of Health and Population, Yemen.

### DNA extraction

One or two discs (6 mm diameter) of 3MM Whatman’s filter paper blood spot (cut by flamed-sterile punch) were used for DNA extraction using a Qiagen blood and tissue kit (QIAGEN, DNeasy® Blood & Tissue Kit, Cat. no. 69506, Germany) according to the manufacturer’s instruction. DNA was eluted using 50 μl AE (10 mM Tris-Cl; 0.5 mM EDTA; pH 9.0) elution buffer (included in the kit) and kept at −20 °C until used.

### Evaluation of HRP2-RDT performance

A malaria-positive blood sample, with a known parasite density (8500 asexual stage/μl) of *P. falciparum* mono-infection, was chosen to evaluate the *CareStart*™ Malaria HRP2-RDT. The blood sample was also checked not to have any sexual stage (gametocytes). Duplicate thick and thin blood films were prepared and stained with Giemsa stain as previously mentioned, and then examined microscopically by two senior malaria microscopists; the average of parasitaemia was recorded. The sample was then diluted in a ratio of 2:3 serial dilutions with healthy human O positive blood group. A total of 12 serial dilutions (labelled 1–12) were prepared in duplicates and the results of asexual-stage parasites density were recorded for each tube [14]. Each tube dilution was then tested for PfHRP2-RDT according to manufacturer instructions.

### Molecular identification and *pfhrp2* sequencing

All DNA from malaria positive samples were confirmed by PCR [15], with *P. falciparum* samples then being considered for *pfhrp2* molecular characterization. Amplification of *hrp2* was carried out in a single-run PCR using a specifically designed oligonucleotide primer pair flanking the region of exon-2 of *pfhrp2* gene [16]. A 50 μL reaction mixture was made up containing 10 μM of each forward (PfHRP2-F 5′-TGTGTAGCAAAAATGCAAAAGG-3′) and reverse primer (PfHRP2-R 5′ TTAATGGCGTAGGCAATGTG-3′), along with 20 μL ExPrime Taq Premix ready-mix PCR reagent (Genet Bio, Korea) and 2 μL of the DNA extract. The amplification thermal conditions were initiated with DNA denaturation at 95 °C for 5 min, followed by 40 cycles of (95 °C/ 30 s, 57 °C/ 40 s and 72 °C/ 90 s) and a single extension step at 72 °C for 10 min. All PCR amplification reactions were amplified using thermal cycler (MyCycler- BioRad, Hercules, USA). Genomic DNA of *P. falciparum* lines 3D7 (MRA-102G), Dd2 (MRA-150G) and HB3 (MRA-155G) provided by Malaria Research and Reference Reagent Resource Centre (MR4), ATCC®, Manassas, VA, USA were used as positive and negative controls for PCR amplification of HRP2 and HRP3. 3D7 was used as a positive control for both HRP2 and HRP3, and Dd2 (a laboratory line known to lack *pfhrp2*) and HB3 (a laboratory line known to lack *pfhrp3*) were used as negative controls for HRP2 and HRP3 respectively [17, 18].

The PCR products were then analysed using agarose-gel electrophoresis. Ten microliters of each amplicon was loaded into a 1.5 % agarose gel and run in a TAE buffer (Tris acetate EDTA), stained with SYBR® safe DNA gel stain (Invitrogen, USA). The fragments size was visualized under UV compared to 100 bp DNA ladder.

The amplicons were sent for purification and sequencing, each amplicon was subjected to sequencing using the same forward and reverse primers as were used during PCR amplification. Both forward and reverse sequences were aligned using BioEdit Sequence Alignment Editor Software (version 7.1.9) and then translated into corresponding amino acids. Each sequence of amino acid repeats was identified and given a code from Type 1 to Type 14 based on the motif being repeated [19].

### Data analysis

The performance of RDT was calculated based on the following indicators: sensitivity, specificity, positive predictive value (PPV) and negative predictive values (NPV) which were calculated with their corresponding 95 % confidence intervals (CI) using Medcalc® online calculator. Moreover, Kappa statistics were used to assess the agreement between RDT and microscopy. This was calculated in IBM SPSS Statistics, version 18.0 (IBM Corporation, NY, USA) by creating a 2 × 2 contingency table. A *P*-value of < 0.05 was considered significant.

## Results

Of the 622 screened individuals, 188 (30.2 %) were positive for malaria by the *CareStart*™ Malaria HRP2-RDT. Of these 188 RDT-positive, only 171 (91.0 %) were confirmed microscopically by detecting either asexual or sexual stages or both. On the other hand, 18 (2.9 %) microscopy-positive individuals were found to be negative with RDT. Similarly, 17 (2.7 %) RDT-positive cases were found to be negative with microscopy. Based on microscopy, the detection rate was the highest in Khamis Bani Saad (39.4 %) followed by Bajil (23.5 %), while Al-Marawiah has the lowest (5.3 %). The level of asexual *P. falciparum* parasitaemia in positive cases ranged from 40 to 55,555 parasites/μl of blood with a geometric mean of 5261 parasites/μl. High parasitaemic individuals (parasite count ≥ 10,000 parasites/μl of blood) represented 17.9 %, while 41.9 % and 40.2 % of the malaria-positive individuals had moderate and low parasitaemia respectively.

The overall sensitivity and specificity of the used *CareStart*™ malaria HRP2-RDT was rated as 90.5 % (95 % CI = 85.4, 94.3) and 96.1 % (95 % CI = 93.8, 97.7), respectively (Table 1). The PPV and NPV were 91.0 % (95 % CI = 85.9, 94.6) and 95.9 % (95 % CI = 93.5, 97.5), respectively. The agreement between the microscopy and HRP2-RDT was statistically significant by Kappa (K = 0.867; *P* < 0.001). In the same vein, HRP2-RDT was examined against serial dilutions of a blood sample with known *P. falciparum* parasite density. The test revealed a high detection rate of *CareStart*™ malaria HRP2-RDT for falciparum malaria isolates; 10 out of the 12 tubes with parasitaemia levels ranged from 8500 to 147 asexual stage/μl were found positive (visible clear band), with a faint band appearing for tube no.11 (98 parasites/μl). The last tube, no. 12, with parasitaemia of 65 parasites/μl was found negative by HRP2-RDT (Table 2 and Additional file 1). With our Yemeni isolates, it was found that performance of RDT increased with parasitaemia level, with the lowest detection rate (44.4 %) reported with parasitaemia levels of 1–100 parasites/μl while it was ≥ 97.0 % with parasitaemia levels of ≥ 1000 parasites/μl. Although most of the negative RDT results were found in samples with low parasitaemia, but negative RDT results were also reported with two samples with moderate and high parasitaemia levels.

**Table 1:** The sensitivity and specificity of PfHRP2-based RDT against the reference technique (microscopy) using samples collected from Hodeidah and Al-Mahwit, Yemen (n = 622)

RDT	Microscopy		
	Negative	Positive	Total
Negative	416	18	434
Positive	17	171	188
Total	433	189	622

Sensitivity: true positive = 171/189 = 90.5 %; false negative error rate = 18/189 = 9.5 %. Specificity: true negative = 416/433 = 96.1 %; false positive error rate = 17/433 = 3.9 %. PPV: 171/188 = 91.0 %; NPV: 416/434 = 95.9 %

**Table 2:** Evaluation of *CareStart*™ PfHRP2-based RDT against serial dilutions of *P. falciparum* parasitaemia

Tube No.	Blood Volume	Diluent (O+ Blood)	Parasite Density	RDT Result
0	Whole Blood	0	8500	Positive
1	200 μl of Tube No. 0	100 μl	5666	Positive
2	200 μl of Tube No. 1	100 μl	3777	Positive
3	200 μl of Tube No. 2	100 μl	2518	Positive
4	200 μl of Tube No. 3	100 μl	1679	Positive
5	200 μl of Tube No. 4	100 μl	1119	Positive
6	200 μl of Tube No. 5	100 μl	746	Positive
7	200 μl of Tube No. 6	100 μl	497	Positive
8	200 μl of Tube No. 7	100 μl	331	Positive
9	200 μl of Tube No. 8	100 μl	221	Positive
10	200 μl of Tube No. 9	100 μl	147	Positive
11	200 μl of Tube No. 10	100 μl	98	Positive
12	200 μl of Tube No. 11	100 μl	65	Negative

Overall, 180/189 cases were successfully amplified for *pfhrp2* and yielded good and quality sequences, and were therefore subjected to amino acid repeat type analysis. The sequence lengths of the isolates were found to vary, ranging from 477 to 879 bp (giving proteins of 159–293 amino acids), with a sequence of 540 bp (180 amino acids) being the most frequent genotype (43.9 %). All isolates sequences were found to be similar in that they started with either a single or multiple Type 1 (AHHAHHVAD), and ended with a single Type 12 repeat (AHHAAAHHEAATH). Overall, Type 1 (AHHAHHVAD), Type 2 (AHHAHHAAD), Type 6 (AHHATD), Type 7 (AHHAAD), Type 10 (AHHAAAHHATD) and Type 12 (AHHAAAHHEAATH) were found in all sequenced isolates. On the other hand, type 9 (AAY) and Type 11 (AHN) were totally absent in all isolates collected from both governorates.

Other amino acid repeat types were varied in their frequencies. Type 8 (AHHAAY), Type 5 (AHHAHHASD) and Type 4 (AHH) ranged between moderate and low, with percentages of 49.4 %, 47.2 % and 15.6 % respectively. Moreover, Type 13 (AHHASD) and Type 14 (AHHAHHATD) were rarely reported in the present study (5.6 % and 2.8 %, respectively). Further, the mean numbers for 2 and 7 repeat types in *pfhrp2* were the highest among the 12 types detected by this study (mean = 9.85 and 4.97, respectively). No association was found between HRP2 repeats and age, sex and districts of participants as well as parasitaemia level. The frequency and mean number of PfHRP2 repeat types from *P. falciparum* isolates from Hodeidah and Al-Mahwit, Yemen are shown in Table 3.

**Table 3:** Frequency of PfHRP-2 repeat types from *P. falciparum* isolates from Hodeidah and Al-Mahwit, Yemen

Type	Amino acids sequence	No. of repeats		Mean^a^	Frequency	
		minimum	maximum		n	%
1	AHHAHHVAD	1	5	3.01	180	100
2	AHHAHHAAD	6	18	9.85	180	100
3	AHHAHHAAY	0	2	1.23	177	98.3
4	AHH	0	2	0.19	28	15.6
5	AHHAHHASD	0	2	0.49	85	47.2
6	AHHATD	2	8	4.11	180	100
7	AHHAAD	3	10	4.97	180	100
8	AHHAAY	0	2	0.52	89	49.4
9	AAY	0	0	0.00	0	0
10	AHHAAAHHATD	1	2	1.25	180	100
11	AHN	0	0	0.00	0	0
12	AHHAAAHHEAATH	1	1	1.00	180	100
13	AHHASD	0	1	0.05	10	5.6
14	AHHAHHATD	0	1	0.03	5	2.8

^a^Average number of amino acid repeat types in the relevant isolates (n)

Interestingly, one isolate with low parasitaemia (184 parasites/μl) was found to be positive by both *CareStart*™ malaria HRP2-RDT and microscopy but it was PCR-negative for *pfhrp2* gene. DNA of this isolate was processed for further PCR confirmation using three PCR protocols that aimed at amplifying *P. falciparum 18SrRNA* [15], *pfhrp2* and *pfhrp3* genes [16]. The DNA was successfully amplified for *18SrRNA* and *pfhrp3* genes while it was confirmed negative for *pfhrp2* gene.

## Discussion

Many rapid diagnostic tests (RDTs) are available to detect different malaria-specific antigens. Most RDTs detect *P. falciparum* specific proteins; either histidine-rich protein 2 (PfHRP2) or *P. falciparum* lactate dehydrogenase (PfLDH), while others can recognize both *P. falciparum*-specific and pan-specific antigens (aldolase (pALD) and pan-species (pLDH) [20]. Previous studies have reported genetic variation in PfHRP2 (genetic deletions, frame shift mutations or alterations in protein expression), which can affect the sensitivity of HRP2-based RDTs, while no variability was observed for pAldolase or pLDH [21, 22]. The HRP2 of *P. falciparum* is a 2-exons gene connected with an intron, located on chromosome 7 (98671–99734 bp) [23]. The amino acid repeats of *pfhrp2* have been characterized into 14 types, based on amino acid motifs being repeated, with Type 2 and Type 7 having been described as possible epitopes targeted by the monoclonal antibodies used to detect *hrp2* [10, 19, 21, 24]

The present study investigated the genetic variation of PfHRP2 among isolates from Yemen, with a possible predicting effect on the performance of the PfHRP2-based RDTs (CareStart® one-step HRP2 RDT; Access Bio Inc., New Jersey, USA) that are used solely for active case detection (ACD) by the national malaria control programme in Yemen (NMCP). A total of 180 isolates were successfully amplified and sequenced for PfHRP2. The sequence lengths of the sequenced isolates varied from 477 to 879 bp (giving proteins of 159 to 293 amino acids). The variations in the number of total amino acids for *pfhrp2* for the Yemeni isolates were found to be lower than the global variation (187 to 306 amino acids), as reported for isolates from 19 countries in Africa, South America, the Pacific region and Southeast Asia [19]. On the other hand, similar variations were reported in Madagascar (145 to 309 amino acids), while a higher number of variations (157 to 333 amino acids) were reported in isolates collected from six different geographical areas in India [16, 25).

Overall, 12 different amino acid repeat types were identified in Yemeni isolates examined by the present study. Of these, six repeat types (types 1, 2, 6, 7, 10 and 12) were found to be present in all isolates, while type 3 was found in 98.3 % of the isolates. These findings are in agreement with isolates from different countries in Africa, Asia and America [21]. In India, six (types 1, 2, 3, 6, 7 and 11) out of 13 identified types of amino acid repeats were detected in all the examined *P. falciparum* isolates [25]. On the other hand, only four types were found in over 98 % of the Senegalese isolates from Dakar [10, 11]. Our study further showed that repeat types 9 (AAY) and 11 (AHN) were totally absent, a result which has been previously reported in isolates obtained from Senegal, Mali, Uganda and Madagascar [10, 11, 16].

We found that all of the amplified isolates (100 %) started with a type 1 repeat (AHHAHHVAD), with either a single or multiple of 2 to 5 copies, before ending with a single Type 12 repeat (AHHAAAHHEAATH). Many previous studies in Africa and Asia showed that all *pfhrp2* sequences begin with a type 1 repeat and concluded with a type 12 repeat [11, 16, 19, 25]. However, a previous study from Dakar, Senegal on 122 sequenced *P. falciparum* isolates had detected three isolates for which *pfhrp2* protein sequences did not begin with a type 1 repeat, and only one isolate found in that study possessed a sequence ending in a type 12 repeat [10].

The present study also showed that the existence of other repeat types in Yemeni isolates varied from moderate (types 8; 49.4 % and 5; 47.2 %) to low (type 4; 15.6 %). Moreover, other types were found only rarely (types 13; 5.6 % and 14; 2.8 %). Our findings are consistent with a previous report on Senegalese *P. falciparum* isolates [10]. By contrast, repeat types 11 and 14 were not detected in isolates from Senegal, Mali and Uganda, while type 4 repeats were detected in all of the isolates [11]. Furthermore, Ugandan isolates were found to be different in that they all contained type 8 (100 %), and lacked type 5 repeats [11]. Moreover, the number of each motif and the total number of repeats within *pfhrp2* vary considerably between countries and within the same country [16, 19]. Yemeni isolates showed high to moderate numbers of the repeat types 2, 7, 6 and 1 within *pfhrp2* (means: 9.85, 4.97, 4.11 and 3.01, respectively) which is higher than what it has been reported in Thailand, the Philippines, Madagascar, Papua New Guinea and South America [16, 19, 26].

Our findings revealed that the HRP2-based RDT possessed good sensitivity; a low parasitaemia level of 139 asexual stages/μl was strongly detectable by RDT. On the other hand, parasitaemia levels below 100 parasites/μl were found either weakly detectable (98 parasites/μl) or totally not detected (65 parasites/μl). These results correspond to RDT results on the Yemeni isolates as we found that detection rate increased with parasitaemia level. However, low detection rate (44.4 %) was reported with samples of very low parasitaemia (i.e., < 100 parasites/μl), as well as two samples (positive with microscopy and pfhrp2-PCR) with moderate and high parasitaemia were found negative. These are in agreement with previous studies which revealed that most of the RDT show excellent detection rates for *P. falciparum* at a parasitaemia greater than 500 parasites/μl, with most of the variation reported at relatively low-level parasitaemia [7, 10, 21]. Moreover, RDT negative results were reported with samples of high-level parasitaemia [27, 28]. This variation could be attributed to either RDT device-related factors such as poor manufacture and deterioration of the device or parasite-related factors such as the level of parasitemia, variability in the target epitopes of the parasite antigen, or quantity of parasite antigen produced by the parasite or present in the peripheral blood, or malaria transmission season [7, 19, 29].

Overall, the present study showed that the sensitivity and specificity of the used HRP2-based RDT were high (90.5 % and 96.1 %, respectively). This was supported by a very good level of agreement between the results of microscopy and RDT tests (Kappa = 0.867). The sensitivity of PfHRP2-based RDT from other reports varied worldwide, ranging from > 90 % (high) to 43.7 % (low) compared to the gold standard microscopy and/or PCR [30–34]. Within the same context, a previous study conducted at the eastern part of Yemen during a malaria outbreak aimed to evaluate the accuracy of PfHRP2-based RDT among 25 falciparum malaria patients, revealed a sensitivity, specificity and positive predictive value of 74 %, 94 % and 68 % respectively when compared to the microscopy [35]. However, the small sample size used in the above study, as well as the potential genetic variation in isolates from the eastern part of Yemen collected during an outbreak when compared to the isolates in this study from malaria endemic areas in western Yemen should be taken into consideration.

In the present study, one sample was found positive for falciparum malaria by microscopy and HRP2-RDT while it was negative by PfHRP2-PCR. The DNA quality was confirmed by successful PCR amplification of both falciparum *18S rRNA* and *pfhrp3* genes. Moreover, the HRP2 PCR protocol was tested against falciparum malaria DNA samples with the same and lower parasite densities which all yielded successful amplification for HRP2 gene suggesting *PfHRP2* gene deletion. Due to *pfhrp3* and *pfhrp2* structural homology, *pfhrp3* can cross-react with HRP2-coated antibodies in the RDT [36], and this may explain the false positive PfHRP2-RDT result by our study. On the other hand, *pfhrp2* gene deletion was reported worldwide and more extensively from South America. It was first reported among Peruvian isolates; 41 % of the malaria-microscopically positive isolates have been found negative by RDT, and failed to amplify the *PfHRP2* gene by PCR [17]. Later, studies from Brazil and Peru [22, 37, 38], Mali in Africa [9], and from India in Asia have reported false negative RDT results due to a *pfhrp2* gene deletion [25].

## Conclusions

The findings of this study provide insights into the genetic diversity of *pfhrp2* in *P. falciparum* isolates from Yemen, and reveal that *pfhrp2* is highly polymorphic in these isolates. *CareStart*™ one-step HRP2-based RDT showed high performance especially for those cases with parasitaemia of >100 parasites/μl. The isolates used by this study were from endemic areas of the Tehama region only. Therefore, population-based studies from other endemic areas throughout Yemen are recommended in order to genetically analyze the *P. falciparum* isolates based on PfHRP2, and to accurately determine the prevalence of parasites that cannot be detected using the PfHRP2 RDT.

## Additional file

Additional file 1:
**Performance of** ***CareStart*** **™ malaria HRP2-RDT against serial dilutions of parasite densities.**
