# Supplementary figures and images for: Genetic variation of pfhrp2 in Plasmodium falciparum isolates from Yemen and the performance of HRP2-based malaria rapid diagnostic test
Source: Parasit Vectors. 2015 Jul 22;8:388. doi: 10.1186/s13071-015-1008-x (PMC4511234; doi:10.1186/s13071-015-1008-x)

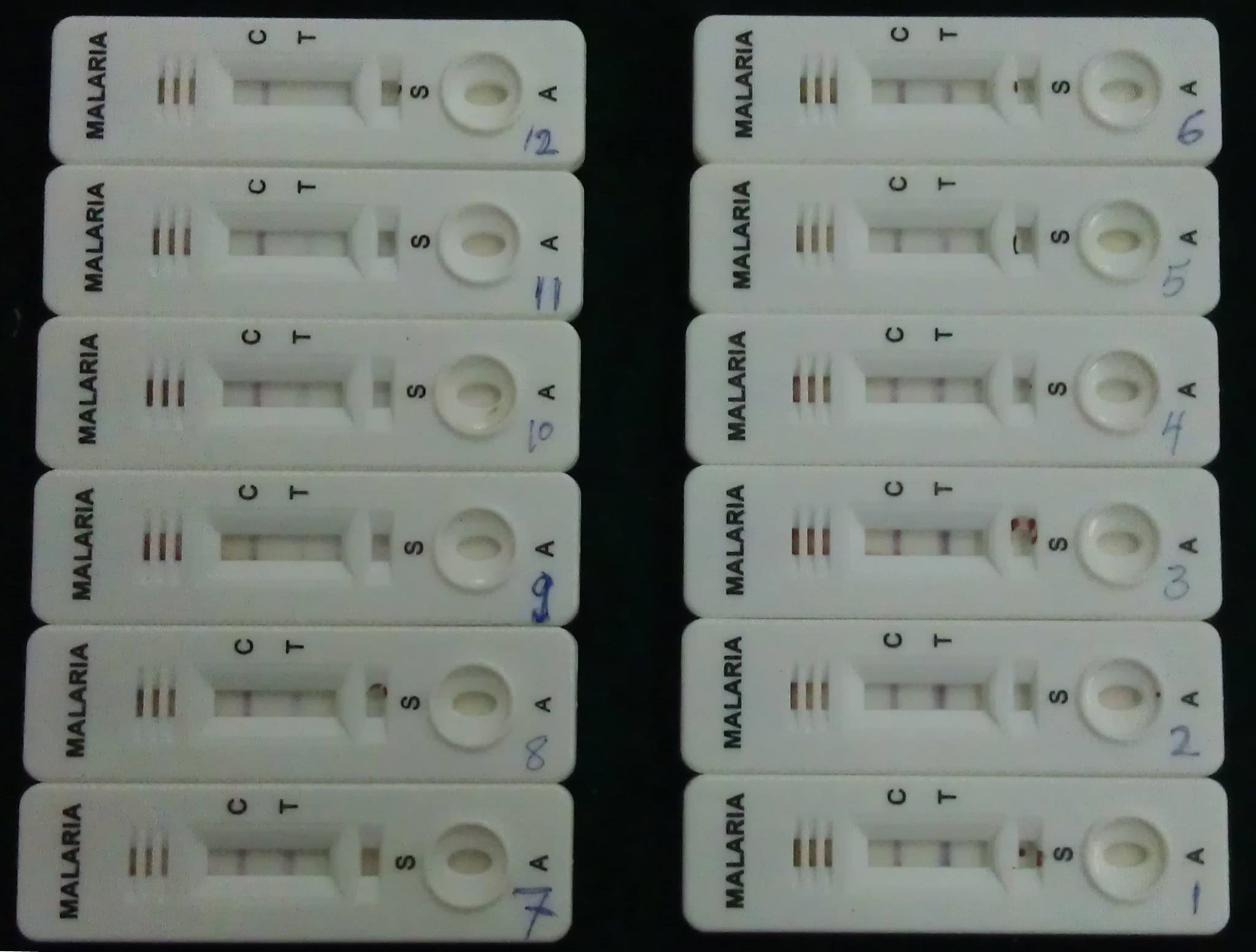

Supplement: Additional file 1: — Performance of CareStart ™ malaria HRP2-RDT against serial dilutions of parasite densities. [file 13071_2015_1008_MOESM1_ESM.tif]
